# A cross-cultural comparison of ethnocentrism and the intercultural willingness to communicate between two collectivistic cultures

**DOI:** 10.1038/s41598-022-21179-3

**Published:** 2022-10-12

**Authors:** Muhammad Yousaf, Muneeb Ahmad, Deqiang Ji, Dianlin Huang, Syed Hassan Raza

**Affiliations:** 1 Center for Media and Communication Studies, University of Gujrat, Gujrat, Pakistan; 2 Chinese Department, National University of Modern Languages, Islamabad, Pakistan; 3 Institute for a Community with Shared Future, Communication University of China, Beijing, China; 4 Institute of Communication Studies, Communication University of China, Beijing, China; 5 Department of Communication Studies, Bahauddin Zakariya University, Multan, Pakistan

**Keywords:** Psychology, Human behaviour

## Abstract

There is a prevalent notion regarding divergence in the extent of ethnocentrism and the intercultural willingness to communicate across cultures. Given this cultural divergence, research is replete with comparative studies of ethnocentrism and the intercultural willingness to communicate between individualistic and collectivistic cultures. However, to our knowledge, a comparison of these crucial cultural tendencies within and their consequences for collectivistic cultures has been overlooked. Thus, this study provides a cross-cultural comparison of ethnocentrism and the intercultural willingness to communicate among university students from two collectivist cultures, i.e., Pakistan and China. The researchers employed a cross-sectional design. A sample of 775 students was collected using a survey technique. The findings show that Pakistani students are more ethnocentric and have a lower intercultural willingness to communicate than Chinese students. Moreover, males were found to be more ethnocentric and less willing to communicate in intercultural settings than females in both countries. These findings validate the notion of ethnocentrism divergence across collectivistic countries and its influence on the intercultural willingness to communicate. Additionally, they demonstrate the role of demographic attributes in evolving ethnocentrism and the intercultural willingness to communicate. Accordingly, these findings also confirm the ecological assumption that contextual factors, such as demographic attributes (e.g., past interactions with foreigners), influence communication schemas. Therefore, concerning its management, these findings suggest that increased people-to-people interactions between the two focal countries can better foster their mutual understanding to reap an increased harvest of the fruits of the Belt and Road Initiative.

## Introduction

Ethnocentrism is a global phenomenon and influences social interaction^1,2^. It has been the source of ethnic strains in different regions, such as South Africa and Lebanon^3^. It is assumed to be a twisted form of racism—a prejudice in individuals’ thinking regarding people they perceive to be the same ethnicity as themselves^4^ and a negative treatment of those who belong to a different ethnicity^5^. However, most ethnocentric research compares individualistic cultures (e.g., the US and Western Europe) with collectivistic cultures (e.g., Korea, Japan and China)^6–8^. It is acknowledged that individualistic (Western) cultures emphasize the content of communications via the explicit and direct meanings of these communications. In contrast, collectivist (Eastern) cultures mainly value the context of communications^9,10^, i.e., meanings are implicit, indirect and context orientated. For that reason, people from collectivistic cultures are relatively more ethnocentric than people from individualistic cultures^11^. Put differently, collectivistic cultures are interdependent, i.e., group decisions are valued more than in individualistic cultures that emphasize personal decisions. Collectivistic individuals are more likely to associate themselves with their cultural group, which corresponds to increased ethnocentrism. Consequently, it is more likely that people show increased prejudice and discrimination in collectivistic cultures than in individualistic cultures^10^. To this end, some scholars have suggested that members of a collectivist culture are anticipated to exhibit distinct ethnocentric attitudes. However, the idea that collectivistic cultures are more ethnocentric than individualistic cultures has not been consistently supported by empirical studies. In this regard, the results show varying trends among both individualistic and collectivistic cultures^8^. For instance, for Korean students^6^ and Chinese students^12^, researchers have reported a lower level of ethnocentrism than among American students, while Japanese students are reported to have a higher level of ethnocentrism than US students^3^. Moreover, international students have scored lower in ethnocentrism from the Malaysian perspective^13^. In contrast, Pakistani university students are more ethnocentric than their Chinese counterparts^14^. These inconsistent findings echo the involvement of various understudied ecological antecedents, which may make the extent of ethnocentrism salient in diverse cultures, irrespective of any patterns narrated in prior cultural models.

Ethnocentrism has an impact on the willingness to communicate, particularly the intercultural willingness to communicate. As a result, it affects the way members of different cultures show the intercultural willingness to communicate. To date, the intercultural willingness to communicate (IWTC) scale has been used by researchers in Australia^15^, Estonia^16^, Micronesia^17^, New Zealand^18^, Russia^19^ Sweden^20^, and, quite recently, in New Zealand^21^. The intercultural willingness to communicate (IWTC) scale is different from the willingness to communicate (WTC) scale. The WTC refers to people’s communication tendencies with friends, colleagues, and strangers. In contrast, the IWTC relates to people’s willingness to be involved in communication encounters with people from different cultures, races, and backgrounds^7^. Ethnocentrism influences the intercultural willingness to communicate among people of different cultures^3,22^. In this context, researchers have found that the more ethnocentric an individual is, the less tendency toward communication the individual shows in intercultural settings^23,24^. Likewise, it has been found that ethnocentrism influences individuals’ understanding of other cultures and upholds their love for their own culture. However, these results are not consistent^25^. Some researchers have found that Korean students are both less ethnocentric and less interculturally willing to communicate than American students^6^. Similarly, it has been concluded that Romanian students have a higher ethnocentric score and lower IWTC than their American counterparts^7^. Moreover, it has found that Pakistani students are more ethnocentric and have less IWTC than Chinese students^26^.

However, it has been found that New Zealand’s management students have a moderate ethnocentric score and that they are also moderate in their intercultural willingness to communicate^21^. Furthermore, the researchers have shown that differences even exist among Asian countries with regard to ethnocentrism and the IWTC in their cross-cultural interactions^6,7^. In a recent study, conducted in Portugal, it has been found that ethnocentrism hinders intercultural communication interactions^27^.

However, despite an increased interest in ethnocentrism and its impact on the IWTC, it is surprising that little empirical research has been conducted in collectivistic cultures while considering possible ecological antecedents. Therefore, what remains to be investigated is how ethnocentrism influences intercultural willingness in collectivistic cultures such as China and Pakistan, where the extended network of family and friends is given much importance. This requires a careful examination of the questions that explain how ecological settings, such as demographic attributes among individuals, influence their interactions while communicating with others that draws from past studies (see for review^28^) that suggest individuals uphold demographic attributes that may influence their patterns of actions based on their ecological environment. Nevertheless, both nations are culturally diverse in terms of their cultural dimensions and cultural orientations (see for review^29^). Correspondingly, these cultural dimensions serve as each society’s collective schemas, guiding the members of a particular society to behave in a specific condition^30^.

Recently, both Pakistan and China have engaged in joint projects ranging from student exchange programs to developmental projects under the Belt & Road Initiative umbrella. Both countries, being collectivistic, provide a unique context for investigating the influence of ethnocentrism and the intercultural willingness to communicate. Thus, it is timely to offer a deeper understanding to policy-makers regarding the intercultural communication relations between the nationals of these two nations. Therefore, this study investigates the extent of ethnocentrism among Pakistani and Chinese students and how it influences their intercultural willingness to communicate in cultural and ecological settings while interacting with outgroups. To the best of our knowledge, there is not a single study that has documented the influence of ethnocentrism on the intercultural willingness to communicate in the context of collectivistic cultures. To address this gap in the literature, this study, therefore, contributes to our understanding of how people from two collectivistic cultures with a different set of values, emotions, and communicative norms interact with one another in intercultural settings.

## Literature review

### Theoretically unpacking the concept of culture

The extensive social sciences literature is categorized into either the ‘essentialist’ or ‘no essentialist’ view of culture^31^. The former is termed positivist, and the latter is labeled ‘interpretive’. Hofstede is considered the proponent of the ‘essentialist’ notion of culture. This view posits that culture within a nation emphasizes categorizing people into different groups based on certain qualities (ibid). Likewise, one’s culture is also differentiated from that of others according to a set of essential qualities. In this view, culture is felt, experienced and seen by other individuals. This promotes stereotyping, i.e., we treat in-groups as superior and outgroups as inferior. In other words, we treat those who come from our own culture different than those who belong to a separate culture. In contrast, the nonessentialist notion of culture treats culture as a moveable entity. In this view, people treat culture as a different thing in different places. The essentialist notion is also called ‘Orientalist’, i.e., people treat cultures as we/them categories. Outgroups are considered inferior and weak, and in-groups are treated favorably and deemed superior^31^. In this study, we have adopted an essentialist notion of culture. The literature is replete with evidence that cultural dimensions influence intercultural interactions; however, this study unswervingly investigates how ethnocentric traits drive communicative actions, such as the willingness to communicate. Compared to past studies that compare the intercultural willingness to communicate between countries based on their individualist vs. collectivist cultural variability, we argue that ethnocentrism can affect the communicative actions of the people in different cultures with the same cultural variability. This is in line with theoretical notions that any ethnocentrism inhibits intercultural communication. Drawing on the orientalist standpoint, the degree of ethnocentric traits determines the evasion that leads to outlining one’s communicative predispositions. In summary, when individuals interact with people from other cultures, they sense dissimilarities, including those in communicative patterns. Most people respond to these differences with an ethnocentric approach, employing their communicative norms that they consider appropriate. As such, when intercultural encounters occur, people apply their own cognitive framework—outlined by their degree of ethnocentrism—and judge any differences, which can lead to an unwillingness to communicate. Further implications of this process are delineated in the next sections.

### Ethnocentrism

Ethnocentrism is a crucial concept for understanding social interactions among individuals in different cultures. Sumner first introduced the term ethnocentrism to the social sciences literature. He defined it as “the technical name for this view of things in which one’s own group is the center of everything, and all others are scaled and rated with reference to it” (p. 13)^32^. That is, one group considers itself superior to other groups. In another study^33^, it has been maintained that ethnocentrism “is our defensive attitudinal tendency to view the values and norms of our culture as superior to other cultures, and we perceive our cultural ways of living as the most reasonable and proper ways to conduct our lives” (p. 157). In this context, some cultures are treated as superior to others. In their study of traditional Chinese culture and art communication in the digital era, researchers observed that treating cultures as ‘us’ and ‘them’ also affects individuals' evaluation of such cultures^34^. This indicates that the attitude of individuals toward a particular culture mediates their evaluation of other cultures. In a similar vein, a positive attitude toward other cultures affects the intercultural communication competence of individuals. This consistent view has been shared in prior studies^1,2,35–37^). These findings suggest the manifestation of ethnocentrism across cultures. Accordingly, everyone is ethnocentric to a certain extent, as ethnocentrism manifests differently based upon individuals’ cultural and ecological education learning. This effect is thus a phenomenon where an individual’s own group is a point of reference for interpreting and evaluating members of other groups or cultures^6,38,39^.

Recently, it has been proposed that “ethnocentrism [is] the belief that one’s own culture is superior to all others. [Where one] view[s] the rest of the world through the narrow lens of one’s own culture” (p. 183)^40^. Additionally, ethnocentrism has mainly been used to study in-group and outgroup attitudes^41,42^. In previous research, scholars^2^ have identified several attitudinal and behavioral characteristics of ethnocentric individuals. Regarding behavioral ethnocentrism, individuals develop good relations with ingroup members but have a sense of competition with outgroup members^2^. The findings have shown that Japanese students are more ethnocentric than American students^1^. Likewise, it was found that Pakistani university students are more ethnocentric than their Chinese counterparts^14^. Thus, ethnocentrism is a crucial barrier to effective communication. Pakistan and China have discrete political and media systems, cultural norms, and values. Building on past cultural models (i.e., Schwartz, Hofstede, and GLOBE), regardless of any similar clusters (collectivism/individualism), all nations have many dissimilarities, such as their orientation toward a specific phenomenon^43^. With respect to collectivism/individualism, Hofstede theoretically identified five dimensions of culture: ‘power distance, uncertainty avoidance, and masculinity/femininity and long W term/short W term orientation’. These cultural dimensions influence the communication of individuals in intercultural contexts along with ethnocentrism (ibid.). Therefore, we argue that both Pakistani and Chinese individuals, while collectivistic, are diversified based on their learned values, including ethnocentric phenomena; thus, in light of the literature, we hypothesize the following:

#### H1

Pakistani students will score significantly higher on ethnocentrism than Chinese students.

### Intercultural willingness to communicate

Communication is a basic human instinct and is central to human interaction; accordingly, it is inevitable for individuals to understand other individuals and perceive cultural variations. Today, humans live in a globalized and rather interdependent world, where the role of intercultural communication has drastically increased. It is an indubitable fact that cultural context influences intercultural communication^44^. Specifically, intercultural communication involves interactions and managing the differences between people from different cultures^45,46^. Intercultural communication also entails “respect for diversity”, which leads to entering into dialog with others and working for “harmony without uniformity”. Such acknowledgment of diversity in intercultural communication is made possible by caring for others’ cultures^47^. This requires justly understanding others’ cultures by utilizing intercultural communication, which can foster individuals to overcome cultural prejudices by engaging with diversity. To this end, the willingness to interact with others is central in ensuring such diversification.

It was found that the willingness to communicate is an “individual’s attitude” when engaging in communication with others^48^. In contrast, it has been suggested that the “intercultural willingness to communicate (IWTC) is defined as one’s predisposition to initiate intercultural communication encounters” (p. 400)^49^. Although the intercultural willingness to communicate (IWTC) seems related to the willingness to communicate (WTC), it is conceptually quite different from the latter^50^. The WTC is related to an individual’s inclination to initiate communication with others when the individual has the freedom to communicate. Put differently, the WTC refers to people’s communication tendencies with friends, colleagues, and strangers. In contrast, the IWTC relates to people’s willingness to be involved in communication encounters with people from different cultures, races, and backgrounds (ibid).

Additionally, ethnocentrism influences intercultural communication. In a study of Japanese and American participants, the more ethnocentric participants had a less empathetic understanding of other cultures, affecting how they interacted with other individuals^3^. Ethnocentrism is also different in various countries, and culture is the main factor that mediates it. In this context, Chinese college students have been shown to be less ethnocentric and have less IWTC than their American counterparts, who are more ethnocentric and have greater IWTC^12^. However, another study has shown that Romanian college students scored significantly higher on the ethnocentric scale and lower on the IWTC scale than their American counterparts^7^. Additionally, it has been reported that Korean college students have significantly lower scores on both the ethnocentric and the IWTC scales than American students^6^. In the Iranian context, it was concluded that ethnocentrism influences the intercultural willingness to communicate between both English and non-English major students^51^. In a more recent study, it was concluded that a higher level of ethnocentrism corresponds to a lower level of the intercultural willingness to communicate among Chinese and Indian undergraduate students studying at a private Malaysian university^52^.

Moreover, some studies of individualistic cultures have explored the IWTC among management students in New Zealand^21^. However, these studies have focused on the individualistic–collectivistic culture dichotomy. To our knowledge, there is no accessible study that has compared ethnocentrism and the intercultural willingness to communicate among respondents from collectivistic cultures.

Although the Chinese and Pakistani cultures are similar to the Japanese and Korean cultures because both fall into the category of collectivistic cultures, both Korean and Japanese participants can vary in their degree of ethnocentrism. Although Chinese and Pakistani cultures are collectivistic, they have many dissimilarities. For example, the shared cultural attributes of individuals in both nations and their tendencies toward collectivism are quite different. For example, Pakistan is scored notably higher than China on the collectivism dimension by Hofstede^29^. Another difference, as narrated above, is their diverse shared cultural attributes, which imply many variances in a given attitude. Thus, there will be a different level of ethnocentrism among participants from these countries; consequently, this will affect the intercultural willingness to communicate. Despite their collectivistic cultures, Pakistan and China share many dissimilarities, ranging from their media systems and political systems to their cultural norms. There should be dissimilarities among people in terms of their willingness to communicate in different cultures due to their disparate norms, values, and communication practices^49^. Therefore, we hypothesize the following:

#### H2

There is a difference in the level of their predisposition toward the intercultural willingness to communicate between Pakistani and Chinese students.

### Influence of ethnocentrism on the predisposition toward the intercultural willingness to communicate

Culture and communication are mutually supportive; one’s level of ethnocentrism affects an individual’s intercultural willingness to communicate with people from other cultures. In this vein, “there is not one aspect of human life that is not touched and altered by culture” (p. 14)^53^. This discussion classifies culture into high-context and low-context. In the former, communication is very explicit, and its meanings are shared by society members; in the latter, communication is implicit, and detailed information, including context, is needed to delineate a message. Asian countries likely hold high-context cultural tendencies. High-context cultures are found in countries such as Korea, Japan, China, and Pakistan; on the other hand, low-context cultures are found in countries similar to the USA and Germany. For instance, individuals from low‐context cultures are more social and confrontation-avoiding than those from high-context cultures^54^. As a result, a greater extent of ethnocentrism is probable within high-context cultures and serves as a mechanism for deciphering cultural differences. Put differently, ethnocentrism affects our understanding of other cultures and influences people’s willingness to communicate with others.

Two communication predispositions, ethnocentrism and the intercultural willingness to communicate, influence individuals’ intent toward intercultural interactions with people from different cultural backgrounds^6^. Thus, ethnocentrism leads to a lack of the intercultural willingness to communicate, which results in cultural conflict. In this context, knowledge of communication predispositions, such as ethnocentrism, helps identify the factors responsible for creating cultural conflict between two cultures^3^. Consequently, it also facilitates adopting effective communication strategies to address a conflict between individuals from two different cultures.

Although ethnocentrism is an individual disposition, it varies from culture to culture and is primarily contextual and cultural. In this regard, ethnocentrism has both negative and positive characteristics. Furthermore, the literature has suggested that members of collective cultures follow in-group authority, are eager to uphold the veracity of their in-group, and are reluctant to collaborate with people from outgroups^3^. Therefore, people in such cultures are expected to be more ethnocentric and to have less willingness to communicate. Moreover, ethnocentric people tend to foster supportive relationships with people belonging to their in-group while being contentious toward and possibly reluctant to cooperate with outgroup members (ibid). Therefore, ethnocentrism is largely considered an adverse trait, associated with intercultural communication. In this scenario, ethnocentrism stems from the ambiguity that can diminish the intercultural willingness to communicate^7,12^. For instance, individuals perceive a higher extent of ambiguity when communicating with outgroup members (e.g., strangers) than with members of their ingroup. Therefore, ethnocentrism-driven intercultural communicative anxiety can prevent individuals from communicating effectively. It can promote putting ‘patriotism’ before one’s own group interests and act as a communication barrier between people from different cultures and backgrounds^22^. Hence, ethnocentrism affects people’s attitudes toward one another in addition to their communication behaviors when they interact with one another in intercultural settings. In light of this literature, we therefore propose our third hypothesis:

#### H3

There is a negative influence of ethnocentrism on the predisposition toward the intercultural willingness to communicate between Pakistani and Chinese students.

### Influence of demographics on ethnocentrism and the intercultural willingness to communicate

Past research has identified that regardless of cultural dissimilarities among cultures, demographic attributes are vital in predicting several predispositions and behavioral outcomes^55^. These demographic attributes and other sociocultural factors, such as norms or beliefs, provide an ecological environment to an individual in a given culture^56^. In turn, individuals learn and groom themselves within this ecological environment^28,43^. For example, people learn acceptable behaviors (i.e., norms), which are regulated by the social institutions available to them in such ecological settings. On the other hand, demographics also expose people to diverse ecological settings, allowing them to learn differently, even within a culture^57^. Hence, each demographic segment (men/women) of a particular culture has a different socialization based on the ecological resources provided to its members^58^. For example, Pakistani women are guided by their family system (ecological resource) concerning how to interact/communicate when encountering men. It is possible that these demographic attributes and ecological settings among diverse nations enable different viewpoints about different actions.

Accordingly, actions and attitudes, such as ethnocentrism and the intercultural willingness to communicate, are certainly influenced by demographic attributes. Thus, demographic variables such as gender, past interactions with foreigners, and background (urban or rural) influence the attitude of respondents. For instance, recent studies^55^ have reported that gender significantly influences ethnocentrism and the intercultural willingness of respondents in intercultural settings. Likewise, a respondent’s background also plays a significant role in his or her ethnocentric score and, consequently, intercultural willingness to communicate. In addition, the experience of interactions with foreigners is another variable that affects ethnocentrism and the intercultural willingness to communicate. The socioeconomic status and gender of a respondent influence his or her academic performance^59^. Therefore, in the context of this study, we assume that the gender, past experience of interactions with foreigners, and rural or urban background of a respondent influence his or her ethnocentrism and intercultural willingness to communicate. Thus, we hypothesize the following:

#### H4

Based on their demographic features such as gender, foreign interactions, and urban or rural background, there is a difference in the influence of ethnocentrism on the predisposition toward the IWTC between Pakistani and Chinese students.

## Research design

### Participants and data collection

In this study, we used a cross-sectional design vis-à-vis the survey method to conduct a cross-cultural comparison of ethnocentrism and the intercultural willingness to communicate between Pakistani and Chinese students. Two samples were purposively chosen from a leading university in Pakistan and in China. The aim of selecting a purposive sample from these two universities was to represent two well-known institutes of communication studies in each country. Students from all parts of these countries select these respective universities for majoring in communications. Thus, considering the nature and significance of this study, the researchers chose the purposive sample of students majoring in communication in both universities. In addition to other places for interaction, university life offers unique opportunities for students worldwide to interact with others^60^. Therefore, exploring ethnocentrism and the IWTC with university students seemed more practical and provided a heterogeneous sample. The students studying at these two universities are almost representative of the total student population majoring in communications. In total, 788 respondents completed the self-report survey questionnaire. After dropping 13 redundant responses, the sample consisted of 775 respondents. In this final sample, seven respondents did not report their gender, 33 did not mention their age, 21 omitted any information about traveling abroad, 27 did not give information regarding their residence, and 18 did not provide any information about their interactions with foreigners.

#### Pakistani sample

A self-report survey questionnaire with demographic variables was administered to Pakistani students enrolled in the communications program at the University of Punjab, Lahore-Pakistan. The survey was in the English language, and the survey instrument included the ethnocentrism and IWTC scales. The Pakistani sample consisted of 387 respondents, of which 167 were males and 217 were females. The respondents’ ages ranged from 18 to 39 years (M = 22.86, SD = 2.3). Eighty-two students had traveled abroad, whereas 291 had never traveled abroad. Ninety-three students came from rural areas, and 282 came from urban backgrounds. Two hundred forty-one students reported that they had experienced interactions with foreigners, and 132 had not had any interactions with foreigners.

#### Chinese sample

The Chinese sample comprised 388 students—74 males and 310 females—enrolled in a communications program. The Chinese respondents’ ages ranged from 17 to 39 years (M = 21.40, SD = 2.8). One hundred seven students had traveled abroad, and 274 had never went abroad. Eighty-four reported a rural background, while 289 were from urban areas. Three hundred nineteen had experience interacting with foreigners, whereas 65 had never interacted with foreigners. For Chinese students, the English version of the revised Generalized Ethnocentrism Scale was translated into Chinese by two native doctoral students enrolled in the communications major. Any discrepancies in their translations were discussed and resolved.

### Measurement of variables

#### Ethnocentrism

To obtain their ethnocentrism score, participants were administered the 22-item revised Generalized Ethnocentrism Scale (GENE). Of its 22 items, 15 are scored to obtain an ethnocentrism score. This 22-item scale is a Likert-type response scale, ranging from strongly disagree = 1 to strongly agree = 5. The ethnocentrism scale has good internal consistency. The higher the score of respondents on the ethnocentrism scale, the higher their ethnocentrism is. For instance, for American and Romanian participants, the Cronbach’s alpha coefficient was previously reported to be 0.90 and 0.81, respectively^7^. In this study, the reliability of the 15-item scale for Chinese participants was 0.86 and 0.81 for Pakistani participants.

#### Intercultural willingness to communicate

The IWTC scale was administered to respondents to measure their intercultural willingness to communicate^49^. The scale consisted of 12 items—half (six) were filler items and half were used to obtain a IWTC score. The IWTC scores ranged from 0 to 100%. A score of 0 means never willing to talk in an intercultural situation, and 100 means always willing to talk. The higher the score respondents have, the greater their intercultural willingness to communicate. For Chinese students, the English version of the IWTC Scale was translated into Chinese by two native doctoral students enrolled in the communications major. Any discrepancies in their translations were discussed and resolved. The Cronbach’s alpha coefficient values for the 6-item IWTC scale in a previous study for Korean and American samples were 0.83 and 0.91, respectively^6^. Likewise, the Cronbach’s alpha coefficient of the 6-item intercultural-willingness-to-communicate scale was 0.90 for an American sample in a study where that of the Romanian participants was 0.81^7^. In the current study, the reliabilities of the 6-item IWTC scale for the Pakistani and Chinese samples were 0.83 and 0.91, respectively.

#### Demographic variables

Drawing on previous studies suggesting the potential role of demographic attributes in predicting ethnocentrism and the intercultural willingness to communicate, this study used three demographic attributes, namely, gender, past interaction with foreigners, and background (urban or rural).

## Results

### Descriptive analysis

Initially, we performed descriptive analysis to test the normality of the data by observing the outliers and histograms that indicated a normal distribution of data across both samples. Table 1 illustrates the mean and standard deviation of the IWTC and ET separately for both samples. In addition, bivariate correlation analysis was performed, which revealed that all variables were significantly correlated across both samples (see Table 3). After normality testing, the study performed confirmatory factor analysis (CFA).

**Table 1:** Descriptive analysis and Pearson correlation statistics.

Variables	Pakistan			China			Pakistan		China	
	M	SD	α	M	SD	Α	ET	IWTC	ET	IWTC
ET	38.49	6.8	0.81	35.73	5.5	0.86	1		1	
IWTC	267.41	160.06	0.91	299.41	136.24	0.83	34	1	21	1

### Confirmatory factor analysis (CFA) and validity

The study used the multigroup methodical approach, which suggests analyzing group differences^61,62^. These differences were examined by CFA for the identification of the invariance and factor loadings. This approach is useful for determining the measurement equivalence of how particular factors remain the same when explaining their parent variables in different cultural settings by constraining and unconstricting the paths. The results of the multigroup CFA reveal that the comparison of both models showed no significant differences, and thus, invariance was verified. Furthermore, the results of the CFA of the Chinese sample (n = 387) reveal that after deleting four items, the third, sixth, and seventh items of ethnocentrism and the second item of IWTC, all other items had loadings better than the suggested cutoff value (0.6)^63,64^. The recommended cutoff criterion for the goodness of fit measures is that the value of × 2/df should be within the range of 1 to 5. It is also recommended to attain at least five indices other than chi-square threshold values that may be employed separately to evaluate model fit. These include baselines and indices such as CFI, TLI and IFI and GFI ≥ 0. 90. For RMSEA and SRMR, values of 0.01, 0.05 and 0.08 indicate outstanding, decent and average fit, respectively, which imply a satisfactory fit. The measurement model solution revealed fit statistics for this research as follows: x^2^/df = 2.72; SRMR = 0.04; RMSEA = 0.043; GFI = 0.92; CFI = 0.95; IFI = 0.97 and TLI = 0.96.

Additionally, the models were tested for discriminant and convergent validity via the factor loadings. Using HTMT analysis, composite reliability and average variance extracted values were examined (see Table 2), and they met the threshold values suggested in the literature^63,65^. The loadings of the factors are given in Table 3.

**Table 2:** Convergent and discriminant validity of the ET and IC constructs.

	CR	AVE	Pak		CR	AVE	China	
			ET	IWTC			ET	IWTC
ET	0.96	0.66	(0.81)		0.95	0.64	(0.80)	
IWTC	0.95	0.63	0.27*	(0.79)	0.88	0.59	0.42*	(0.77)

*CR* composite reliability, *AVE* average variance extracted, and values in parentheses are the square root of AVE.

*Significant at *p* < 0.001.

**Table 3:** Loadings of (ET) and (IWTC) constructs among Pakistani and Chinese samples.

Items	China	Pakistan
**Ethnocentrism**		
Item ET1	0.79	0.81
Item ET2	0.82	0.89
Item ET3	0.44*	0.53*
Item ET4	0.91	0.87
Item ET5	0.88	0.76
Item ET6	0.51*	0.25*
Item ET7	0.52*	0.36*
Item ET8	0.81	0.78
Item ET9	0.69	0.75
Item ET10	0.87	0.77
Item ET11	0.72	0.89
Item ET12	0.85	0.79
Item ET13	0.73	0.68
Item ET14	0.89	0.71
Item ET15	0.81	0.87
**Intercultural willingness to communicate**		
IWTC1	0.87	0.75
IWTC2	0.62*	0.32*
IWTC3	0.72	0.68
IWTC4	0.83	0.81
IWTC5	0.67	0.84
IWTC6	0.62	0.76

*p* < 0.01.

*Items were deleted due to low loading or to attain M.I.

### Hypothesis testing

Independent samples t tests were used to test three hypotheses (H1, H2, and H4). The independent samples t test was conducted to compare the ethnocentrism score for Pakistani and Chinese students; its results indicate that there is a significant difference between Pakistani (M = 38.49, SD = 6.8) and Chinese students (M = 35.73, SD = 5.5; t (773) = 6.17, p = 0.000, two-tailed). Similarly, the results of an independent samples t test comparing the intercultural willingness to communicate between the two cultures show that Chinese students have a higher intercultural willingness to communicate score (M = 299.41, SD = 136.24) than Pakistani students (M = 267.41, SD = 160.06, t (771) =  − 2.99, p = 0.003, two-tailed). Moreover, an independent-sample t test between gender and the ethnocentric scores indicated that male participants (M = 38.00, SD = 6.34) are more ethnocentric than female participants (M = 36.66, SD = 6.35; t (766) = 2.73, p = 0.007, two-tailed). Likewise, an independent samples t test for the IWTC between the two samples indicated that male respondents (M = 259.89, SD = 143.06) have less intercultural willingness to communicate than females (M = 294.25, SD = 150.60, t (764) =  − 2.98, p = 0.003, two-tailed). In other words, female participants are more willing to communicate with people from different cultures. When we compared within-sample differences for gender, we found that within Pakistan, there is no significant difference for the ethnocentrism score between males (M = 38.5, SD = 6.90) and females (M = 38.48), SD = 6.94, t (382), = 0.05, p = 0.96, two-tailed). However, within the Chinese sample, we found that males (M = 36.84, SD = 4.67) are more ethnocentric than females (M = 35.38, SD = 5.56, t (382) = 2.08, p = 0.04, two-tailed). For both samples, there were no significant differences in the IWTC between males and females. We did not find a significant difference in the ethnocentrism score for those 189 respondents who reported that they had traveled abroad (M = 36.44, SD = 6.39) or for the 565 who had not traveled abroad (M = 37.29, SD = 6.36, t (752) = − 1.95, p = 0.11, two-tailed). Similarly, no significant difference was found for the intercultural willingness to communicate among those who had traveled abroad (M = 298.86.SD = 145.9) and those who had not traveled abroad (280.60, SD = 150.69, t (750) = 1.45, p = 0.148 (two-tailed).

An independent samples t test for rural students showed no significant difference in the ethnocentrism score between rural (M = 37.16, SD = 6.32) and urban students M = 37.16, SD = 6.36, t (746) = 0.004, p = 0.99, two-tailed). Our independent samples t test for the IWTC of urban and rural students showed that urban students (M = 292.43, SD = 149.4) scored significantly higher than rural students (M = 263.44, SD = 145.84, t (744) = − 2.26, p = 0.02, two-tailed). Hence, urban students have a greater IWTC than their rural counterparts. When we compared those students who had experience interacting with foreigners to those who did not, we found a significant difference in ethnocentrism between the former (M = 36.36, SD = 6.11) and the latter (M = 39.20, SD = 6.64, t (755) = − 5.47, p = 0.000, two-tailed). However, there was no significant difference in the IWTC of students who had such interactions (M = 289.44, SD = 145.31) and those who did not (M = 269.09, SD = 156.89, t (753) = 1.65, p = 0.09 (two-tailed). An independent samples t test comparison between undergraduate and postgraduate students regarding their ethnocentrism score showed a significant difference. The undergraduate students (M = 37.7761, SD = 6.56) were more ethnocentric than the postgraduate students (M = 36.4241, SD = 6.13, t (773) = 2.96, p = 0.003 (two-tailed). For the IWTC analysis, there was also a significant difference. The undergraduates (M = 297.53, SD = 155.26) had a greater tendency toward the intercultural willingness to communicate than the postgraduate students (M = 269.01, SD = 141.75, t (771) = 2.66, p = 0.008 (two-tailed).

**Figure 1:**
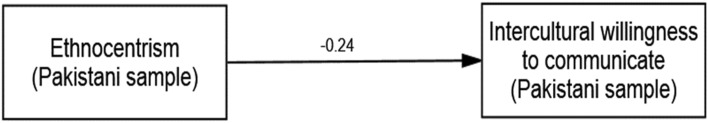
Structural model (Pakistani sample).

Moreover, to validate Hypothesis H3, we constructed two structural models for each country’s sample, i.e., one for China and one for Pakistan (see Fig. 1). This approach permitted us to detect the all-inclusive suitability of the proposed models for both samples and whether the data could validate the structural models^63,66^. The results of the commonly used fit indices revealed each model’s goodness of fit (Table 4).

**Table 4:** Confirmatory factor analysis (structural models).

Model	× 2	df	× 2/df	GFI	IFI	CFI	RMSEA
Pakistan	2417.54	902	2.68	0.93	0.97	0.95	0.042
China	2267.65	895	2.53	0.95	0.98	0.97	0.034

**Figure 2:**
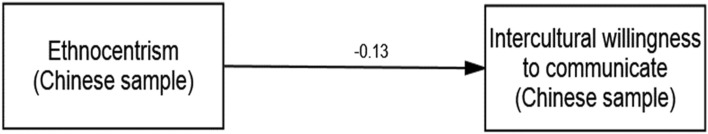
Structural model (Chinese sample).

The results for our test of H3 illustrate that ethnocentrism negatively affected the predisposition to intercultural competence (= − 0.24, p = 0.05) in the Pakistani sample and negatively affected the predisposition to intercultural competence (= − 0.13, p = 0.04) in the Chinese sample (see Figs. 1, 2).

However, these results supported H3 regarding the Pakistani sample due to its high score on the ethnocentrism scale. Hence, ethnocentrism more negatively affected the predisposition to intercultural competence among the Pakistani sample than among the Chinese sample.

## Discussion

Our first hypothesis (H1) posits that Pakistani students would have more ethnocentric scores than their Chinese counterparts. In this study, the Pakistani students scored significantly higher on the Ethnocentrism (GENE) scale than the Chinese students. Therefore, Hypothesis 1 is supported. Our second hypothesis suggests that there is a difference between the predisposition to the intercultural willingness to communicate between Pakistani and Chinese students. This hypothesis is also supported. As Pakistani students are more ethnocentric, they consequently have less intercultural willingness to communicate than Chinese students, who are less ethnocentric and have a greater tendency toward the intercultural willingness to communicate. These findings validate the notion presented in existing cultural theories, such as Hofstede’s^29^, i.e., national culture drives individuals’ schemas of actions.

Similarly, a plausible explanation could be drawn from a national culture; that is, regardless of, e.g., a similar Asian context, there are certain cultural dissimilarities across cultures. For example, individuals living together amid shared cultural characteristics, such as norms, regulate their actions and tendencies to react in a situation^42^. Furthermore, the ecological environment where people socialize affects their behavioral patterns^56^. Likewise, the Chinese ethnic group has a higher education level. They are more self-centered and less cooperative, but they are also more diligent, tolerant and easy-going, respecting other ethnic minorities and cherishing family values^67^. Therefore, Chinese students are less ethnocentric and more willing to communicate in intercultural settings. In contrast, Pakistan is a diverse country, housing religious and ethnic minorities; there, the religious element is more dominant than the cultural element.

Consequently, Pakistani students are more ethnocentric and less interculturally eager to interact. One of the likely reasons for this greater ethnocentrism and lesser intercultural eagerness to engage in intercultural interactions is the religious socialization of the students. They are more reserved and less willing to engage in intercultural communication with people with another origin, culture, and values. The males in both samples are more ethnocentric than females and consequently less willing to engage in intercultural communication.

These findings support those of previous research^6,12^. These findings also support the rich body of communication studies, suggesting that females are more open and relation oriented during communications^68^. This research line can explain why females are less ethnocentric and more willing to communicate interculturally than their male counterparts. Thus, China’s rise as an economic player on the global stage and its subsequent integration with the world amid increasing cross-cultural exchanges provide Chinese students more opportunities for interaction and communication in different intercultural settings with people from different countries with different cultures and political and social values. Therefore, their lower level of ethnocentrism and greater intercultural willingness to communicate than Pakistani students makes sense and is not surprising.

The students with an urban background were also more willing to communicate in intercultural situations than those who reported rural backgrounds. In both countries, contemporary urban cities such as Beijing, Shanghai, Lahore, and Islamabad provide more cultural, educational, and communication-based opportunities for students than their rural counterparts. This enhances their competence and cultural communication abilities in different situations with different people from diverse backgrounds. Therefore, their greater intercultural willingness to communicate in different intercultural circumstances is also reasonable. Additionally, the students who had experience interacting with foreigners were less ethnocentric than those who did not. Consequently, we conclude that ethnocentrism influences the intercultural willingness to communicate. Chinese students are less ethnocentric and more willing to communicate interculturally, whereas Pakistani students are more ethnocentric and have a lower tendency toward the intercultural willingness to communicate.

### Implications of intercultural understanding for a community of shared interests

Despite multidimensional cultural differences, ranging from political systems and media systems to religious and cultural taboos, a better intercultural understanding can be achieved through the effective use of intercultural communication between two nations. It is, therefore, essential to recognize that intercultural understanding can be achieved by introducing new courses in curricula and by giving students assignments that have intercultural communication dimensions in colleges and universities. This will provide them with an opportunity to become familiar with sensitive issues related to culture, ethnicity, and religion. This sheds light on the significance of intercultural dialog for increasing intercultural understanding and harmony between people from both China and Pakistan. It is pertinent for achieving the shared political, economic, and diplomatic goals and objectives of both countries. The more we have such interactions, the more we can understand and celebrate our differences, consequently reducing intercultural differences. In this regard, “although the challenges of an increasingly diverse world are great, the benefits are even greater” (p. 4)^69^. To reap the benefits of this increasingly diverse and integrated world and prepare themselves for better intercultural communication, Chinese and Pakistani students should equip themselves by actively engaging in intercultural communication interactions. These intercultural engagements can be beneficial. In this context, a significant decrease in students’ pre- and postscores of ethnocentrism is observed in a service-learning project via a different cultural context^70^. Likewise, increased intercultural interactions between Chinese and Pakistani politicians, media practitioners, businessmen, and students could foster better intercultural understandings by avoiding sensitivities and appreciating common grounds while celebrating differences to further strengthen the ties between the two countries. Moreover, these findings could be useful in business interactions, educational settings, and media engagements, improving the understanding of political norms, values and cultures to create a harmonious environment to reap an increased harvest of the fruits of the Belt & Road Initiative. In addition, both countries can include more content about each other in their educational curricula to enhance the familiarity of their people with the relevant politics, religion, ethics, and economics. In this regard, evidence from a different context has shown that collaborative learning between students in America and New Zealand via email was beneficial for developing their intercultural competence and improving their understanding of each other^71^. Similarly, China and Pakistan can enhance the intercultural competence of their peoples to create a community of shared interests.

### Policy implications

In light of the findings of this article, the researchers suggest that some practical steps should be taken by relevant stakeholders. At a practical level, exchanges between the two countries should be encouraged to enable first-hand experiences and a better and deeper understanding of their media and political systems and cultural sensitivities. The relevant stakeholders should take into consideration the lack of proper media representation in each country among their respective media outlets. Although after the launch of the China-Pakistan Economic Corridor (CPEC), the presence of each country in the other’s media has significantly improved, there is a need for more media content based on shared objectives, cultural dimensions, and festivals to foster better intercultural communication and understanding among ordinary people, government officials, and nongovernmental organizations. In this regard, more cultural exchanges among politicians, media practitioners, businesspeople, cultural industry professionals, movie makers, and students is a prerequisite for promoting better awareness of both countries’ cultures, norms, customs, and values. Accordingly, scholars have suggested that increased first-hand experiences and interactions are essential to understanding the relevant issues and sensitivities while strengthening the relationship between countries^72^. These interactions can improve cultural understandings among the public in both countries. Moreover, this enhances empathetic understanding, which is effective for better communication in different cultures where communication practices are supposed to be different.

To bridge the gender ethnocentrism gap on a more general level, more cross-cultural communication courses should be introduced in colleges and universities, providing more opportunities and a better understanding of different cultures and their norms for multicultural coexistence and appreciation. Such courses can allow students to orient themselves with other cultures, appreciate similarities, and celebrate differences. This engagement can help students appreciate the sensitivities of other cultures and better manage intercultural conflicts in organizational and economic contexts. In summary, in a globalized world, intercultural competency is a broad-ranging and critical issue, affecting communication within interpersonal contact and in the domain of business. There is a long-standing debate regarding the important phenomenon of how cultural divergence serves as a key factor in barriers to cultural competence. This paper thus provides empirical evidence and validates the notion of cultural divergence within rarely studied collectivistic cultures. This evidence can be a starting point to consider cultural tendencies while planning for cultural competence among the two focal nations, which have recently initiated business and cultural ties. For example, other intercultural interactions under the Belt and Road Initiative could also aid businesspersons, officials and interpersonal communication in both countries if the relevant authorities take the abovementioned steps. Hence, we could reap a larger harvest of the fruits of a common and shared destiny based on mutual respect and goals under the vision of the ‘Belt and Road Initiative’ vision by promoting intercultural harmony and mutual understanding between these brotherly and friendly countries.

### Strengths and limitations

The strength of this study is its consideration of the native cultures of both countries, i.e., China and Pakistan. This has resulted to some extent in a more representative perspective of both China and Pakistan than is readily available to international students. However, this research has some limitations as well. First, the sample of this study was limited to university students, and the data reported here are based on a self-reported survey of the respondents. Although the sample size is large and has statistical significance, it is not strictly random. Therefore, the findings should be generalized with caution.

On the other hand, selecting university students helps control for and minimize the variance and diversity present in these two countries’ populations. Second, much of the research on intercultural communication has centered on cultural dimensions, such as high and low context culture communication and individualistic and collectivistic cultures. We suggest that scholars look beyond the dichotomy of individualistic and collectivistic cultures^73^ and explore the influence of cultural background and other demographic variables on ethnocentrism and, consequently, the intercultural willingness to communicate between different individuals. Such an approach can help us better understand the communication traits and predispositions that affect effective cross-cultural communication between individuals with different cultural backgrounds. Finally, there is a need to explore ethnocentrism and its influence on the intercultural willingness to communicate within both developed and developing countries with different demographic variables.

### Institutional review board statement

The study was conducted according to the guidelines of the Declaration of Helsinki and approved by the Institutional Research Ethical Committee of CMCS, University of Gujrat, Gujrat, 50700, Pakistan, Letter No. UOG/CMCS/2020/180.

### Informed consent statement

Informed consent was obtained from all subjects involved in the study.

## Data Availability

The data that support the findings of this study are available from the corresponding author upon reasonable request due to ethical and privacy restrictions.
